# Potential for Rabies Control through Dog Vaccination in Wildlife-Abundant Communities of Tanzania

**DOI:** 10.1371/journal.pntd.0001796

**Published:** 2012-08-21

**Authors:** Meagan C. Fitzpatrick, Katie Hampson, Sarah Cleaveland, Lauren Ancel Meyers, Jeffrey P. Townsend, Alison P. Galvani

**Affiliations:** 1 Division of Epidemiology of Microbial Diseases, Department of Epidemiology and Public Health, Yale University, New Haven, Connecticut, United States of America; 2 Boyd Orr Centre for Population and Ecosystem Health, Institute for Biodiversity, Animal Health and Comparative Medicine, College of Medical, Veterinary and Life Sciences, University of Glasgow, Glasgow, United Kingdom; 3 Section of Integrative Biology, The University of Texas at Austin, Austin, Texas, United States of America; 4 Santa Fe Institute, Santa Fe, New Mexico, United States of America; 5 Program in Computational Biology and Bioinformatics, Department of Ecology and Evolutionary Biology, Yale University, New Haven, Connecticut, United States of America; George Washington University, United States of America

## Abstract

Canine vaccination has been successful in controlling rabies in diverse settings worldwide. However, concerns remain that coverage levels which have previously been sufficient might be insufficient in systems where transmission occurs both between and within populations of domestic dogs and other carnivores. To evaluate the effectiveness of vaccination targeted at domestic dogs when wildlife also contributes to transmission, we applied a next-generation matrix model based on contract tracing data from the Ngorongoro and Serengeti Districts in northwest Tanzania. We calculated corresponding values of *R*_0_, and determined, for policy purposes, the probabilities that various annual vaccination targets would control the disease, taking into account the empirical uncertainty in our field data. We found that transition rate estimates and corresponding probabilities of vaccination-based control indicate that rabies transmission in this region is driven by transmission within domestic dogs. Different patterns of rabies transmission between the two districts exist, with wildlife playing a more important part in Ngorongoro and leading to higher recommended coverage levels in that district. Nonetheless, our findings indicate that an annual dog vaccination campaign achieving the WHO-recommended target of 70% will control rabies in both districts with a high level of certainty. Our results support the feasibility of controlling rabies in Tanzania through dog vaccination.

## Author Summary

The vaccination of dogs against rabies is an effective method for the control of the disease and its transmission to humans in many locations, including in North America and Europe. However, vaccination coverage for only dogs might be insufficient in countries such as Tanzania, where wildlife has been shown to also carry and transmit rabies. To address this concern, we developed a model parameterized with field data on rabies in dogs and wildlife from two districts of Tanzania, Ngorongoro and Serengeti. Our study found significant variation in the patterns of rabies transmission between the two districts, with wildlife playing a more important part in rabies transmission in Ngorongoro. Based on these findings, a higher vaccination coverage level against rabies is necessary in Ngorongoro more than in Serengeti, although it is still possible to achieve the required level of vaccination coverage for Ngorongoro through dog vaccination alone. An annual dog vaccination campaign at the WHO-recommended target of 70% would control rabies in both districts.

## Introduction

Rabies is a viral encephalitic disease, transmitted to humans primarily from rabid animals. Once symptoms appear, human rabies is almost inevitably fatal [1], leading to an estimated 55,000 human deaths each year [2]. Over 7.5 million post-exposure human vaccines are distributed annually [2], with an economic burden of US$ 1 billion worldwide [3].

Domestic dogs account for more than 95% of human exposures. Consequently, canine vaccination has the potential to concomitantly prevent disease in humans [4]–[7]. Where domestic dogs are the reservoir hosts, canine vaccination has been shown to be an effective control strategy in many parts of the world [6]. For example, domestic dog vaccination has led to the elimination of canine rabies in Western Europe and the US [8], [9] and to widespread control of the disease in Latin America [10]. Nonetheless, there remains skepticism regarding the degree to which large-scale dog vaccination campaigns can control or eliminate dog rabies in the presence of abundant wildlife host species, and concern that coverage levels which have historically been sufficient for rabies control elsewhere might be insufficient in these settings [7].

Previous theoretical models of rabies transmission have contributed predictions of disease dynamics and control. Notably, a general model of rabies in dog populations predicted that a constantly maintained canine vaccination coverage of 70% should control the disease [11], while a model parameterized for Kenya recommended 70% coverage targets for annual campaigns [12]. Other models have considered alternate strategies to specifically control rabies in wildlife populations, such as the use of oral vaccination baits or culling [13]–[16]. However, none of these models have explicitly evaluated how transmission in hosts other than domestic dogs might impact the success of mass canine vaccination, or how it might affect the necessary vaccine coverage level.

We evaluate the feasibility of rabies control in regions with abundant wildlife populations, using a next-generation model that incorporates rabies contact tracing data from Ngorongoro and Serengeti Districts in Northern Tanzania. Traditionally, calculations to find the proportion of a population that must be vaccinated to control rabies assume that vaccination is uniformly applied across all hosts, and do not account for control effort targeted at a subset of host types. To estimate the required coverage level for a successful rabies vaccination campaign directed at domestic dogs in the presence of other host species, we adapted a multi-host transmission model proposed by Roberts and Heesterbeek [17]. Our results indicate that canine vaccination is indeed a feasible strategy to control rabies in a multi-host system. Additionally, we provide estimates for the confidence of program success at a range of vaccination coverage levels for both Tanzanian districts.

## Methods

### Model

We modeled rabies transmission in our system using the next-generation matrix approach [17], for which we defined two host types: (1) domestic dogs and (2) other carnivores, including domestic cats and a variety of wildlife species. Dogs are considered as a separate host type because they are the target of vaccination campaigns in Tanzania. The number of transmission events from wildlife into the dog population plays a role in determining how much vaccination will be required in dogs, but the species from which transmission occurs does not. Thus, we combined wildlife species together as a second host type. In this context, the term “generation” refers to reproduction of the infection rather than reproduction of the host.

The matrix describing the reproduction of the infection is
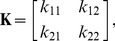
(1)where *k_ij_* denotes the expected number of secondary cases in host type *i* that are the result of a single case in host type j. The dominant eigenvalue of this next-generation matrix is *R*_0_, the basic reproduction number [18], defined for a single-host disease system as the average number of new infections caused by one typical infection in a completely susceptible population. In this multi-host context, we focus instead on the type-reproduction number *T_1_*, which denotes the expected cumulative number of infected dogs in the chain of transmission that begins with a single rabid dog, disallowing the reproduction of secondary infections in dogs [17]. For the two-host system described by the matrix **K**,
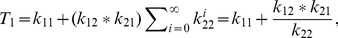
(2)where the first term captures direct transmission from the initial dog to other dogs, and the second term captures infection of dogs by wildlife as the outbreak spreads among wildlife. Equally targeting all host types, the minimum vaccination fraction that will prevent a disease outbreak (p_c_) is 1−1/*R*_0_. However, in practice, rabies vaccination campaigns commonly target dogs exclusively. In this case, T_1_ replaces *R*_0_ in the calculation of p_c_, and 1−1/T_1_ yields the minimum vaccination fraction for domestic dogs that should prevent an epidemic in the wider host population [17].

### Data

Contact tracing data were collected in Ngorongoro and Serengeti Districts for every rabies case that was detected from January 2002 through December 2006 [19], [20]. Serengeti is inhabited by agro-pastoralist communities with relatively dense populations of domestic dogs (9.4 dogs/km^2^). The Serengeti ecosystem is also home to abundant and diverse populations of wildlife. Ngorongoro is inhabited by pastoralist communities and low density domestic dog populations (1.4 dogs/km^2^). We analyzed the two districts separately because of these distinctions and because of differences in the logistics for dog vaccination campaigns [21].

The methods and results of this data collection have been reported elsewhere [19], [20]. Briefly, every incident reported either as an animal-bite injury to a hospital or dispensary or a suspect rabid animal through a livestock office or community-based surveillance study was investigated by the research team. To determine the source of exposure and subsequent contacts, villagers were interviewed by veterinary officers, local leaders, and livestock field officers in attendance to facilitate the development of an active surveillance network. The location of the incident was recorded, as well as whether the animal disappeared, was killed, was restrained, or died “naturally” of rabies. Cases were diagnosed through both epidemiological and clinical criteria. Brain samples were collected whenever possible, but the majority of cases were suspected rather than confirmed. When tested, more than 75% of samples led to confirmed rabies diagnoses, indicating the robustness of the clinical and epidemiological criteria [20]. As a result of the identification of these initial rabies cases, the subsequent exhaustive tracing of linked suspect cases, and the resulting active surveillance network, many more cases were detected than would be reported through traditional surveillance channels. Although we are unable to determine exactly what proportion of rabies cases were detected using this method, we assume a high probability of detection given the consistency between various population and individual-level epidemiological parameters previously estimated from this data [19], [20].

Information regarding 107 suspect rabies cases in domestic dogs was recorded for Ngorongoro. Of these, 20 infectious dogs were restrained or killed (19%). Data for 24 suspect cases in other species were recorded. In Serengeti, there was information for 778 suspect cases in domestic dogs, of which 127 dogs were restrained or killed (16%). Data for 92 suspect cases in other species were recorded including cases in domestic cats, civets, genets, hyenas, honey badgers, jackals, leopards, white-tailed mongooses, and wildebeest. To facilitate our analysis of these systems without intervention, cases in which the rabid dog was killed or restrained were removed from parameter estimation, although not from the construction of epidemic trees (see below).

Contact tracing generated detailed spatiotemporal data on the timing and location of cases and in many instances identified whom transmitted to whom. A previously described tree-building algorithm [19], [20] was used to probabilistically infer transmission links between identified cases based on the geographic distance and timing between cases and the spatial infection kernel and generation interval distribution from these natural infections (Figure S1). This algorithm was used to address concerns about underreporting of transmission involving wild animals, for which we were unable to identify ancestor-descendant relationships. We constructed 1000 probabilistic epidemic trees using the spatiotemporal data to infer connections for each identified case. The log-likelihood of every tree fell within twice the value of the log-likelihood of the most likely tree, so all trees were accepted as valid possible representations of the true epidemic.

### Parameterization and Uncertainty

We categorized each transmission event into one of the types described by the four *k_ij_* elements in the matrix **K**. Additionally, the prevalence of vaccinated domestic dogs within the surrounding community at the time of each transmission event was modeled using an exponential decay function that incorporated the number of dogs that had been vaccinated during the most recent campaign in that village, the elapsed time since that campaign, and domestic dog vital rates as reported in [19]. The value of *k_ij_* was estimated by modeling the number of secondary infections (of type i) as a Poisson process at rate λ. The expected number of secondary infections (of type *i*) is then equal to λ. To account for the reduced number of transmission events per rabid animal in a population where some dogs are vaccinated, and to standardize all cases to the population without vaccination, we modulated the Poisson process by the probability p that the bitten dog was susceptible. The estimated vaccination coverage in the domestic dog population at the time of the case was then 1-p.

The likelihood of a particular λ, l_λ_, is estimated through the summation of the probability of that λ value given the number of secondary infections resulting from a single rabid animal, n_i_, and the vaccination coverage level associated with that event, p_i_, over all rabies events in the dataset [22]. The calculation for this likelihood is given by
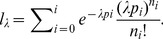
(3)The time interval for the process during which secondary infections are accumulated is considered to be a single infectious period, or 1.

To find the most likely *k_ij_* values, we first chose a single random tree and assessed the likelihood of its data given values of λ between 0.01 and 2, at increments of 0.0001, in a case wherein vaccine coverage was not considered (i.e., *p* = 1). Then, initializing λ at the value which had maximized the likelihood, we used a Markov chain Monte-Carlo random walk algorithm to find the most likely values of λ, corresponding to *k_ij_*, given both our case information and our estimated vaccination coverage levels. We initiated a jump of random size and direction (from a triangular distribution with a range of −1 to 1) from our starting λ to a new potential λ value. Additionally, we randomly chose a new epidemic tree to provide the data for assessing the likelihood at each jump step. When the newly chosen value for λ yielded a greater likelihood than the starting point, it was accepted as a data point and the new starting place. When this value yielded a lower likelihood, it was accepted with a probability equal to the ratio of the two likelihoods [23]. After a “burn-in” of 10,000 points to allow convergence to a stationary distribution, this procedure was iterated 10,000 times. Examination of output shows convergence well before the end of the “burn-in” period (Figure S2). If the probability of acceptance fell below 15% after the burn-in was completed [24], the size of the jump was reduced and the whole process was repeated. The acceptance ratio for all *k_ij_* parameters fell between 0.15 and 0.41. The recorded points are likely values for each k_ij_. We report the median of the distribution as our point estimate of each parameter.

The 10,000 samples from the *k_ij_* distributions were used to calculate 10,000 values for *T_1_* in each district. We then calculated *p_c_* from each *T_1_* value. This procedure propagated sampling error to yield a distribution for the sustained vaccination coverage level required to prevent an outbreak of rabies. The rabies control programs that have demonstrated success in Tanzania [21], [25] have conducted annual vaccination campaigns. Accordingly we estimated the coverage level that would need to be achieved during a single annual campaign, p_a_, such that vaccination coverage would be sustained above p_c_ until the following year. With an adult dog annual death rate of d = 0.45 for both districts and respective annual growth rates of r = 0.102 and 0.09 for Ngorongoro and Serengeti [19],
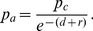
(4)We used equation (4) to generate a *p_a_* value for each *p_c_* value in our distribution. The percentiles of the distribution of p_a_ indicate the level of confidence that a policy maker should have that an annual vaccination campaign will be successful in controlling rabies. Annual campaign targets that were adequate in fewer than 50% of the samples were not considered viable strategies.

## Results

**Figure 1:**
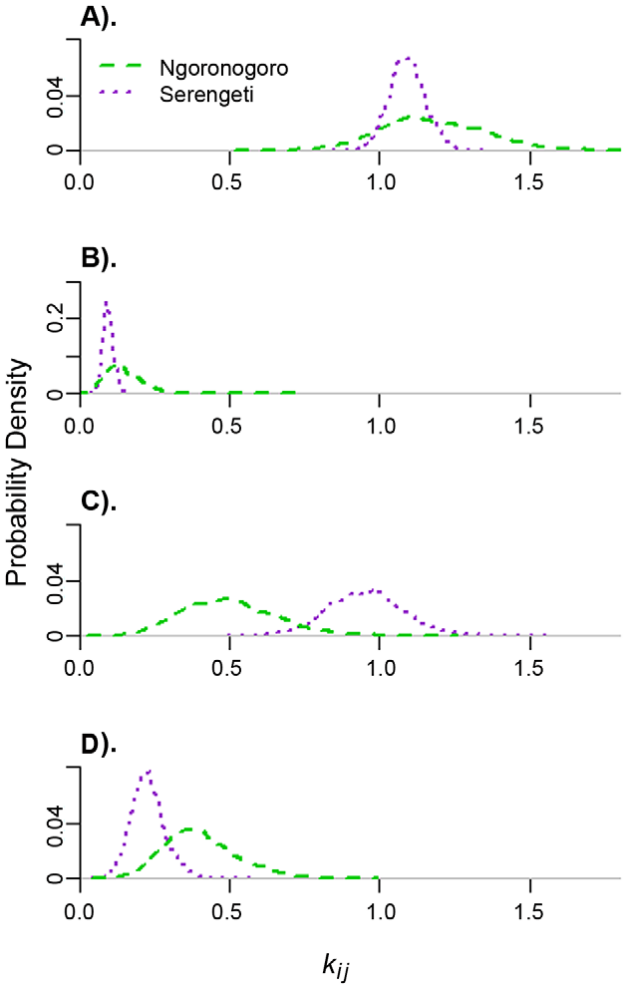
Rabies transmission by host type. Probability densities for each *k_ij_*, or the expected number of secondary infections of type i that result from a single infection of type j. Domestic dogs are host type 1, and all other animals belong to host type 2. (a) *k_11_*, (b) *k_21_*, (c) *k_12_*, (d) *k_22_*.

In our model without human intervention, the maximum likelihood estimate for the expected number of secondary domestic dog infections from a single infected domestic dog was similar for both districts, at 1.16 for Ngorongoro and 1.09 for Serengeti, and was the highest host type-to-host type transmission rate in each district (Table 1, Figure 1). The lowest estimated transmission rate was from infected domestic dogs to other host species, again similar in both districts at 0.13 for Ngorongoro and 0.09 for Serengeti. The maximum likelihood estimate for transmission from infected alternative hosts to domestic dogs in Serengeti was nearly double that for Ngorongoro, at 0.95 and 0.49, respectively (p<0.05). Conversely, the maximum likelihood estimate for transmission within alternative host species in Ngorongoro was estimated to be 70% higher than that in Serengeti, at 0.39 compared to 0.23. There is considerably larger sample variance in all Ngorongoro parameters than in the Serengeti counterparts, due primarily to the much lower number of cases in Ngorongoro. Both distributions of transmission rates among wildlife fall below one. Thus, based on current data, transmission within wildlife is not self-sustaining in either district. *R*_0_ for rabies in the system overall was estimated to be 1.24 for Ngorongoro and 1.18 for Serengeti.

**Table 1:** Estimates of epidemiological parameters.

	*Direction of Infection*	Ngorongoro	Serengeti
***k_11_***	dog to dog	1.16 (0.85–1.54)	1.09 (0.98–1.21)
***k_21_***	dog to other	0.13 (0.05–0.27)	0.09 (0.06–0.13)
***k_12_***	other to dog	0.49 (0.23–0.84)	0.95 (0.71–1.21)
***k_22_***	other to other	0.39 (0.20–0.67)	0.23 (0.13–0.35)
****R***_0_*	basic reproduction number	1.24	1.18
***T_1_***	adj. reproduction number (95% CI)	1.29 (0.96–1.69)	1.21 (1.08–1.35)
***p_c_***	critical coverage required (95% CL)	23% (39%)	17% (25%)
***p_a_***	annual coverage required (95% CL)	39% (67%)	30% (42%)

The values for *k_ij_*, refer to the maximum likelihood estimate for the expected number of secondary cases of rabies of type *i* as a result of a single case in type *j*, in two districts of Tanzania. *R*_0_ is calculated as the Eigenvalue of the next generation matrix *K* for each district. For *T_1_*, the 95% confidence interval is noted in parentheses, and for *p_c_* and *p_a_* the 95% confidence limit is given.

**Figure 2:**
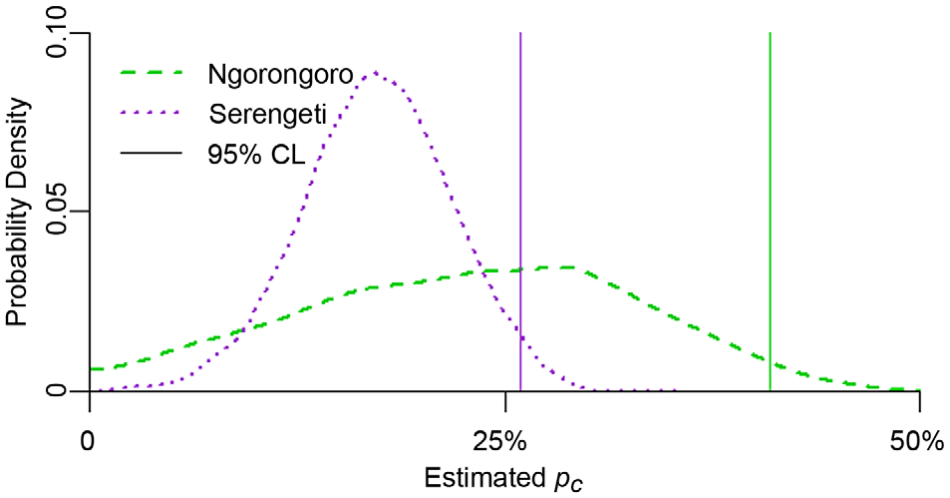
Probability densities for *p_c_*, the minimum vaccination fraction, in Ngorongoro and Serengeti. Solid vertical lines indicate the coverage level at which there is a 95% confidence that rabies will be controlled.

**Figure 3:**
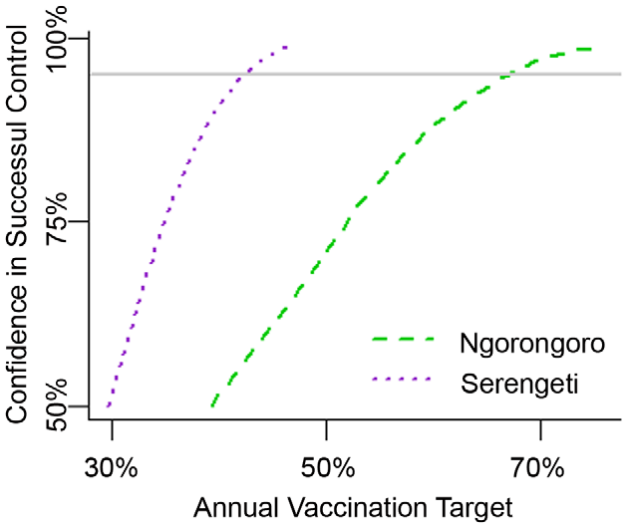
Confidence that vaccination targets for annual campaigns will control rabies. Confidence that a vaccination coverage level in domestic dogs achieved during an annual campaign will control rabies for the duration of one year, in Ngorongoro and Serengeti. The horizontal line represents a 95% confidence that rabies will be controlled at the corresponding level of annual vaccination.

The maximum likelihood estimate for the critical level of vaccine coverage (*p_c_*) was estimated to be slightly lower in Serengeti than in Ngorongoro (17% compared to 23%)(Table 1, Figure 2), as was the continuous level of coverage needed to attain 95% confidence of rabies epidemic prevention (25% compared to 39% coverage). Likewise, the median estimated annual target for Serengeti and the 95% confidence target were both lower than that for Ngorongoro (30% compared to 39%, and 42% compared to 67% target coverage, Figure 3). There is an approximately 5% chance that *p_c_* and *p_a_* could fall below 0 in Ngorongoro (Figure 2), indicating a very low probability that rabies epidemics in this district will die out without intervention. The probability of fade-out without vaccine intervention was also negligible in Serengeti.

## Discussion

We have shown that annual canine vaccination campaigns achieving 67% coverage in Ngorongoro and 42% coverage in Serengeti should be sufficient to control rabies outbreaks with 95% confidence. These coverage levels are lower than the WHO-recommended annual target of 70% [26]. We focused on annual coverage targets, since rabies vaccination in Tanzania is conducted through annual campaigns and since the WHO target is specified as such. However, we also calculated that 39% and 25% coverage consistently maintained in Ngorongoro and Serengeti, respectively, will control rabies outbreaks with 95% confidence. These estimates of required coverage are much lower than previous recommendations of 70% coverage on a consistent basis [11]. The difference is possibly due to fact that the parameters of the previous study were drawn from Asia and the Americas, whereas our model considers rabies dynamics in sub-Saharan Africa [19]. Our conclusions also suggest that lower coverage levels may be effective than those predicted to be necessary for annual campaigns in Kenya [12]. This is probably related to a high *R*_0_ value (2.44) found in the Kenyan study. These differences may reflect regional differences in rabies transmission, a conclusion supported by the different transmission dynamics found between our two districts. These differences may also reflect the high dog density in the Kenyan study sites, which could provide increased opportunities for disease transmission [13]. Additionally, a re-analysis of the epidemic reported in the Kenyan study resulted in a lowered value of *R*_0_ (1.72, CIs: 1.34–2.18) [19] while the primary: secondary case ratio was based on relatively limited data (44 cases) compared to those from Tanzania (1001 cases) and is likely subject to more stochastic variability. Various methods have been used to provide estimates of *R*_0_ in Tanzania and these *R*_0_ values have been reported between 1.05–1.32 [19]. Worldwide, most estimates of *R*_0_ fall below 1.7 [19], further indicating that the original estimates from Kenya may be anomalously high. None of these aforementioned studies, however, incorporated transmission in species other than dogs, leaving open the question of whether such transmission might make control by canine vaccination either more difficult or impractical. We have demonstrated that canine vaccination at the levels recommended by the WHO will be more than sufficient to control rabies even in the multi-host setting of northwest Tanzania. In addition, we have shown that control of rabies in canine hosts has the indirect benefit of controlling rabies in wildlife.

For simplicity, we used the minimum distinct host-types necessary to describe our system: dogs in one class, and other terrestrial carnivores in another. Further stratification would have been possible, with each species treated as its own class of host. However, vaccination campaigns in Tanzania are directed at dogs alone, and the creation of such distinctions between any other hosts has no impact on the recommended vaccination coverage level in dogs. Additionally, the low sample size for most species in our data sets produces such large uncertainty in our model as to obscure any potentially useful conclusions that a closer examination of wildlife species might bring.

Although rabies transmission in wildlife is not self-sustaining, it does increase overall transmission in the system and causes differences in the transmission dynamics between the two districts. Transmission rates within alternative host species were much higher in Ngorongoro than in Serengeti. Presumably, the higher density of wildlife in Ngorongoro provided a greater abundance of susceptible alternative hosts for transmission events from the typical rabid wild animal. In contrast, wildlife were much more likely to transmit rabies to domestic dogs in Serengeti, probably due to the higher density of domestic dogs in this district. In turn, rabid dogs may be transmitting the disease to new hosts in proportions based more on their socialization than on relative species abundance in the area, resulting in similar rates for transmission from dogs to dogs and from dogs to wildlife in the two districts.

Although this model has demonstrated that relatively low levels of vaccination coverage should be sufficient to control rabies in these populations, we must emphasize the need for annual revaccination of the dog population. Elimination is an unlikely possibility without concerted regional control efforts, as reintroduction events from neighboring endemic areas commonly occur [27]. We also caution against direct extrapolation of these estimates into policy recommendations for target vaccination coverage any lower than 70%, as human health benefits will undoubtedly accrue as outbreaks from these reintroductions are controlled more swiftly. Further study into the costs and benefits to humans of canine vaccination is warranted to clarify this point.

In summary, we have analyzed contact tracing data to demonstrate that a program of canine vaccination has the ability to control rabies in the Ngorongoro and Serengeti districts of Tanzania, even in the presence of transmission within and among other species. We parameterized transmission rates, accounting for sampling uncertainty for rabies in northwest Tanzania. Our analysis provides a framework for accounting for uncertainty in disease transmission among multiple types of hosts, evaluating the effectiveness of rabies control strategies, and guiding policy makers in their control efforts against this devastating disease.

## Supporting Information

Figure S1 **Representation of the Ngorongoro rabies transmission over time.** Black nodes represent rabies cases in dogs, gray nodes represent cases in livestock, and red nodes represent cases in other animals. The vertical axis corresponds to the longitude at which the case was recorded. Black edges correspond to transmission events confirmed during data collection. Solid gray edges represent transmission events identified by construction of the most likely epidemic tree. Dashed gray lines represent other possible transmission events identified through iterated tree construction.
(TIF)

Figure S2 **Transmission parameters converge to stable distributions before the end of the burn-in period.** The value accepted at each of the first 2000 iterations of the MCMC random walk for (a, e) *k_11_*, (b, f) *k_21_*, (c,g) *k_12_*, and (d, h) *k_22_* in (a–d) Ngorongoro and (e–h) Serengeti.
(TIF)
