# Supplementary figures and images for: Potential for Rabies Control through Dog Vaccination in Wildlife-Abundant Communities of Tanzania
Source: PLoS Negl Trop Dis. 2012 Aug 21;6(8):e1796. doi: 10.1371/journal.pntd.0001796 (PMC3424251; doi:10.1371/journal.pntd.0001796)

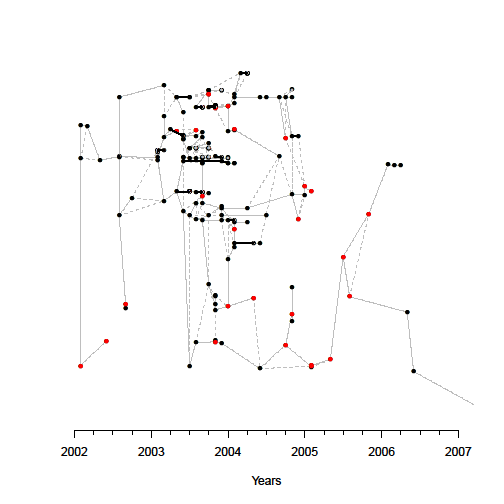

Supplement: Figure S1 — Representation of the Ngorongoro rabies transmission over time. Black nodes represent rabies cases in dogs, gray nodes represent cases in livestock, and red nodes represent cases in other animals. The vertical axis corresponds to the longitude at which the case was recorded. Black edges correspond to transmission events confirmed during data collection. Solid gray edges represent transmission events identified by construction of the most likely epidemic tree. Dashed gray lines represent other possible transmission events identified through iterated tree construction. (TIF) [file pntd.0001796.s001.tif]

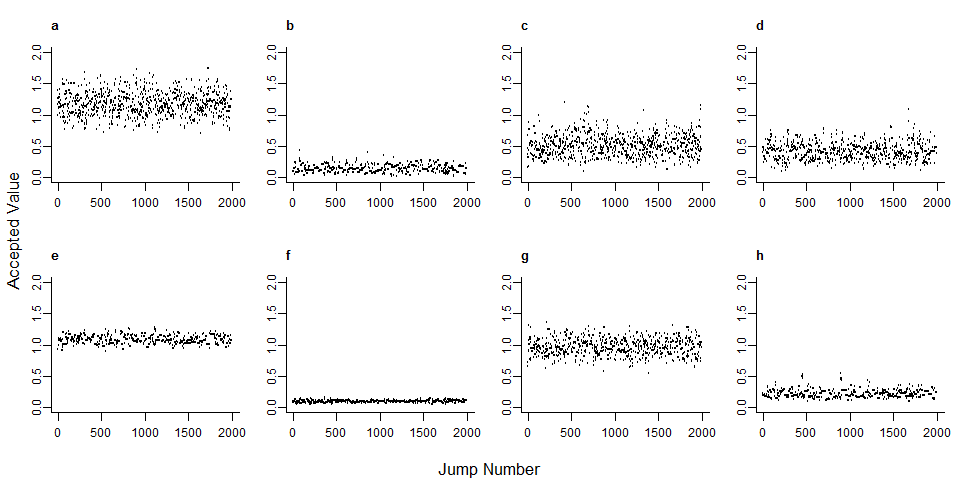

Supplement: Figure S2 — Transmission parameters converge to stable distributions before the end of the burn-in period. The value accepted at each of the first 2000 iterations of the MCMC random walk for (a, e) k11, (b, f) k21, (c,g) k12, and (d, h) k22 in (a–d) Ngorongoro and (e–h) Serengeti. (TIF) [file pntd.0001796.s002.tif]
